# A Multicenter Pilot Randomized Trial of a Lifestyle Intervention to Prevent Type 2 Diabetes in High-Risk Individuals

**DOI:** 10.3390/nu17152518

**Published:** 2025-07-31

**Authors:** Raira Pagano, Thatiane Lopes Valentim Di Paschoale Ostolin, Danielle Cristina Fonseca, Aline Marcadenti, Ana Paula Perillo Ferreira Carvalho, Bernardete Weber, Carla Daltro, Enilda Lara, Fernanda Carneiro Marinho Noleto, Josefina Bressan, Jussara Carnevale de Almeida, Malaine Morais Alves Machado, Marcelo Macedo Rogero, Olivia Garbin Koller, Rita de Cássia Santos Soares, Sônia Lopes Pinto, Viviane Sahade, Cleyton Zanardo de Oliveira, Guilherme William Marcelino, Camila Martins Trevisan, Angela Cristine Bersch-Ferreira

**Affiliations:** 1 BP—A Beneficência Portuguesa de São Paulo—PROADI-SUS, São Paulo 01323-000, SP, Brazildanielle.candian@bp.org.br (D.C.F.); w.bernardete@gmail.com (B.W.);; 2 Hcor Research Institute (IP-Hcor), São Paulo 04004-030, SP, Brazil; amarcaden@hcor.com.br; 3 Graduate Program in Health Sciences (Cardiology), Instituto de Cardiologia/Fundação Universitária de Cardiologia do Rio Grande do Sul (IC/FUC), Porto Alegre 90650-090, RS, Brazil; 4 Graduate Program in Public Health, Faculdade de Saúde Pública da Universidade de São Paulo (FSP-USP), São Paulo 01246-904, SP, Brazil; 5 Unidade de Hipertensão Arterial, Hospital das Clínicas da Universidade Federal de Goiás, Goiânia 74605-020, GO, Brazil; 6 Departamento Ciência da Nutrição, Escola de Nutrição, UFBA—Universidade Federal da Bahia, Salvador 40110-907, BA, Brazil; carlahcdaltro@gmail.com (C.D.);; 7 Independent Researcher, São Paulo 04143-010, SP, Brazil; 8 Programa de Pós Graduação em Ciências da Saúde, Curso de Nutrição, UFT—Universidade Federal do Tocantins, Palmas 77020-021, TO, Brazil; 9 Graduate Program in Nutrition Science, Department of Health and Nutrition, Universidade Federal de Viçosa, Viçosa 36570-900, MG, Brazil; jbrm@ufv.br (J.B.);; 10 Departamento de Nutrição, Faculdade de Medicina, Universidade Federal do Rio Grande do Sul (UFRGS), Porto Alegre 90610-264, RS, Brazil; jcalmeida@hcpa.edu.br; 11 Programa de Pós Graduação em Alimentação, Nutrição e Saude, Programa de Pós Graduação em Ciências Médicas: Endocrinologia, Universidade Federal do Rio Grande do Sul (UFRGS), Porto Alegre 90035-003, RS, Brazil; ogkoller@hcpa.edu.br; 12 Serviço de Nutrição e Dietética, Hospital de Clínicas de Porto Alegre, Porto Alegre 90610-261, RS, Brazil; 13 Department of Nutrition, School of Public Health, University of São Paulo, São Paulo 01246-904, SP, Brazil

**Keywords:** type 2 diabetes prevention, lifestyle intervention, dietary quality, physical activity, BALANCE program, Brazilian public health, randomized controlled trial, feasibility study

## Abstract

Background: Type 2 diabetes (T2D) is a growing public health concern, particularly in low- and middle-income countries. Although prediabetes is a major risk factor for T2D, it remains largely underdiagnosed and untreated. Structured lifestyle interventions have proven effective in preventing diabetes, but their feasibility within the Brazilian public health system remains unclear. Methods: This multicenter pilot randomized controlled trial assessed the feasibility of a culturally adapted lifestyle intervention (PROVEN-DIA) across the five regions of Brazil. A total of 220 adults at high risk for T2D were randomized to an intervention group or a control group (usual care) and followed for three months. Both groups received similar educational content on healthy eating and physical activity, but the intervention group participated in a structured and personalized lifestyle program with regular follow-up sessions. The primary outcome was adherence to dietary recommendations, assessed using the BALANCE Index—a validated dietary score (range: 0–40) based on the Brazilian Cardioprotective Diet that classifies foods into color-coded groups according to nutritional quality—along with engagement in moderate-to-vigorous physical activity (MVPA). Secondary outcomes included diet quality (DQIR), anthropometric and metabolic parameters. Results: Feasibility was demonstrated by a 93.2% retention rate (n = 205). There was no significant difference in the primary outcome (simultaneous improvement in diet and MVPA). However, the PROVEN-DIA group exhibited significantly greater improvements in diet quality, with a 2.8-point increase in the BALANCE Index (vs. 0.5 in the control, *p* = 0.03), and a significant improvement in the DQIR (*p* < 0.001). No significant differences between groups were observed in MVPA, HbA1C, glycaemia, or body weight. Conclusions: The PROVEN-DIA intervention proved feasible within the Brazilian public health context, resulting in significant improvements in dietary quality among individuals at high risk for T2D. A larger trial with longer follow-up is warranted to evaluate its effectiveness in preventing the progression to diabetes. However, to enhance physical activity outcomes, specific adaptations and targeted strategies may be required to better support participant engagement in exercise.

## 1. Introduction

Type 2 diabetes (T2D) is a growing public health concern, particularly in low- and middle-income countries, where its incidence and complications are rising rapidly [1]. In Brazil, the increasing prevalence of T2D has placed a substantial burden on the healthcare system, especially due to its strong association with cardiovascular disease [2]. Prediabetes—a high-risk metabolic condition that precedes the onset of T2D—is also a major concern globally, yet it remains largely underdiagnosed and undertreated in clinical settings [3,4]. In Brazil, population-based data from the National Health Survey (Pesquisa Nacional de Saúde) reported that between 7.5% and 17.5% of adults have prediabetes [5]. This is concerning, as a recent meta-analysis showed that individuals with prediabetes have a significantly higher risk of all-cause mortality and cardiovascular disease compared to those with normal glycemic levels [6]. These findings underscore the importance of early identification and intervention in individuals at high risk for diabetes—even before the disease is fully established.

There is strong evidence that preventing T2D is more effective and less costly than treating the disease [7,8,9]. Since lifestyle plays a central role in the development of T2D—through its influence on modifiable risk factors such as overweight, hypertension, and prediabetes—promoting healthy behaviors is a key public health strategy. The Diabetes Prevention Program (DPP), a landmark randomized trial conducted in the United States, demonstrated that structured lifestyle interventions focused on diet, physical activity, and behavioral change significantly reduce the incidence of T2D among individuals at high risk, both in the short and long term [10,11]. Following the DPP, similar interventions have been successfully adapted and implemented in several countries, including Finland [12,13], the United Kingdom [14], India [15], and China [16]. However, evidence on the feasibility and effectiveness of such programs within the Brazilian public healthcare system remains limited.

Given the structure of Brazil’s public health system—the *Sistema Único de Saúde* (SUS), one of the largest universal healthcare systems in the world, which provides free and comprehensive services to the entire population through a vast primary care network—the lack of culturally adapted diabetes prevention strategies, a lifestyle intervention was developed specifically for the Brazilian context. The PROVEN-DIA Pilot Study aimed to assess the feasibility of this approach. Unlike the DPP, which also tested metformin, PROVEN-DIA focused solely on lifestyle change. This decision was based on its broader health benefits, greater potential for long-term behavior change, and superior cost-effectiveness. Although metformin is inexpensive and widely available in the public system, it may not promote sustained engagement and may cause side effects [8,10,11,17,18]. A cost-utility analysis of the DPP showed that lifestyle intervention delayed diabetes onset more effectively and had a lower cost per quality-adjusted life year than metformin [9]. These findings support the choice of evaluating a lifestyle-based strategy as a stand-alone solution in the Brazilian context. Therefore, the PROVEN-DIA Pilot Study proposed two groups that received guidance based on materials from the Brazilian Ministry of Health to encourage a healthy diet and increased physical activity. However, only the intervention group received this guidance through a structured lifestyle intervention, which consisted of a program with scheduled group and individual sessions, personalized goal setting, follow-up phone calls, and strategies for overcoming barriers. Estimating participants’ adherence to lifestyle changes and demonstrating improvements in diet quality and weekly physical activity time would provide valuable evidence to guide future investments in a potential national program.

Hence, this study presents the results of a multicenter pilot randomized controlled trial designed to evaluate the feasibility of this lifestyle intervention in preventing T2D among high-risk individuals in Brazil. Findings from this pilot trial are intended to inform the design of a larger, definitive study.

## 2. Materials and Methods

### 2.1. Study Design

We conducted a multicenter, open-label, two-arm, parallel-group randomized pilot trial at five centers across Brazil, between May and October 2023 (ClinicalTrials.gov: NCT05689658). The sites were strategically selected to represent the cultural diversity of Brazil’s five regions and included academic institutions with expertise in diabetes care and experience in conducting multicenter randomized trials. The study followed the ethical principles of the Declaration of the Americas and applicable local regulations and was approved by the Research Ethics Committee of the coordinating center, BP—A Beneficência Portuguesa de São Paulo (protocol code CAAE: 65491122.7.1001.5483), alongside all five centers on each Research Ethics Committee. All participants provided written informed consent before enrollment, following a thorough explanation of the study’s objectives, procedures, and potential risks by trained staff. In addition, the study was reported as recommended by CONSORT 2010 statement: extension to randomized pilot and feasibility trials [19], as can be seen in Supplementary Table S1. Similarly, the study flowchart was presented as proposed by the CONSORT statement.

Each site was responsible for promoting the trial and recruiting participants through local media, social networks, and partnerships with health services. Eligible individuals identified during screening were invited for an in-person visit to confirm eligibility and proceed with enrollment.

### 2.2. Participants

Inclusion criteria were body mass index (BMI) higher than 24.9 kg/m^2^, internet access, possession of a personal mobile device, reside near the center, no prior nutrition counseling (within six months prior to the study), and at least one of the following characteristics: fasting glucose levels between 100 and 125 mg/dL; glucose levels 2 h after an oral glucose tolerance test between 140 and 199 mg/dL; HbA1c levels between 5.7% and 6.4%; previously medical diagnosed with gestational diabetes; high risk on the Centers for Disease Control and Prevention (CDC) prediabetes risk test. 

The diabetes risk score used in this study was based on the American Diabetes Association (ADA) and Centers for Disease Control and Prevention (CDC) Risk Test, which itself was adapted from the algorithm developed by Bang et al. [20]. This non-invasive screening tool assigns points to key demographic and clinical variables associated with the risk of developing type 2 diabetes: age, sex assigned at birth, body mass index (BMI) classification, physical activity, history of hypertension, and family history of diabetes. Each factor contributes a specific score (ranging from 0 to 1 point or 0 to 3 points), and the total score ranges from 0 to 10. In our study, individuals scoring 5 points or higher were classified as being at high risk for developing type 2 diabetes and were considered eligible for inclusion.

Exclusion criteria were adults with a confirmed diagnosis of T2D, human immunodeficiency virus antibodies detectable (HIV), previous cardiovascular disease (as stroke or acute myocardial infarction), chronic conditions that interfere in life expectancy or ability to participate in the study, participation in another randomized clinical trial, and pregnancy or breastfeeding women.

### 2.3. Randomization, Blinding and Withdrawal

Participants were randomly assigned in a 1:1 ratio to either the intervention group (Proven-Dia) or the control group (usual treatment [UT]) using the study’s centralized web-based system, REDCap^®^. The allocation sequence was computer-generated using validated software (R statistical software, version 4.2.1), employing block randomization with varying block sizes and stratification by center. Investigators accessed REDCap^®^, completed the electronic case report form (CRF), and confirmed eligibility criteria before randomization was permitted.

Due to the nature of the intervention, blinding was not feasible for participants or study staff. Treatment could be discontinued at any time by participant request, medical decision, or at the discretion of the study team. Participants lost to follow-up were retained in the statistical analysis according to the intention-to-treat principle.

### 2.4. Procedures

Participants were followed for three months. The participants allocated to the UT group underwent an initial face-to-face visit and another follow-up after three months. The UT was standardized according to guidance materials from the Brazilian Ministry of Health, which aimed to enhance healthy diet and promote physical activity. Participants were advised to practice 150 min of moderate-to-vigorous physical activity (MVPA) per week, alongside guidance on adopting a healthy and cardioprotective diet, reducing the intake of ultra-processed foods, as recommended by the Cardioprotective Diet: a manual for primary healthcare professionals [21,22,23]. Additionally, participants received an educational brochure containing all dietary and physical activity guidelines for reference throughout the three-month period.

The Proven-Dia participants were instructed to achieve the same objectives as the UT group. However, these objectives were translated into a program aimed at facilitating and encouraging participants to adhere to these goals: improving the diet quality and promoting physical activity for at least 150 min at moderate to vigorous intensity.

The Proven-Dia comprises six in-person sessions: three group sessions led jointly by the ‘planner’ and the ‘facilitator’, and three individual sessions led solely by the ‘planner’. Additionally, the Program includes five phone calls led by the ‘facilitator’. Each phone-based session followed a structured script designed to reinforce participants’ commitment to their personalized goals, assess progress, identify barriers, and offer motivational support. These calls were led by trained facilitators and occurred weekly between the in-person sessions. They complemented the content of the face-to-face meetings by encouraging adherence and providing tailored guidance based on participants’ individual needs and reported difficulties. Further details on the session plan, group meeting structure, and participant workbook are available in Supplementary Methods S2 (Supplementary Table S4).

The lifestyle change goals were collaboratively determined between planner and participant. This approach ensured that the goals were individualized and personalized, typically comprising three objectives. Two of these goals should focus on diet and physical activity, while the third goal could be either a diet or a physical activity goal. In each follow-up visit, the participants could reassess his improvement and subsequently readjust their goal based on what they deemed feasible. The dietary goals focused on adhering to a healthy and cardioprotective diet, based on the Brazilian Cardioprotective Nutritional Program [23,24] and the Dietary Guide for the Brazilian Population [22]. The physical activity goals were based on the Physical Activity Guide for the Brazilian population [21], aiming to achieve at least 150 min of moderate-to-vigorous physical activity (MVPA).

The Brazilian Cardioprotective Nutritional Program (BALANCE) [23,24] categorizes foods according to the colors and proportional areas of the Brazilian flag (Figure S1), based on nutritional criteria including calorie density, saturated fat, cholesterol, and sodium content. The green group—fruits, vegetables, skimmed milk, and beans—is recommended for frequent consumption. The yellow group includes grains, nuts, and oils, which should be consumed in moderation. The blue group consists mainly of animal proteins, recommended in smaller amounts. The red group, not represented in the national flag, refers to ultra-processed foods and should be avoided. Individualized menus (1400–2400 kcal/day) are designed with specific portions from each group. While originally developed for cardiovascular disease prevention, BALANCE has been adapted to diabetes prevention by capping daily carbohydrate intake at 45–48% (reference value for moderate carbohydrate-restricted diet). Nutritional composition and portion details are available in Supplementary Methods S1 (Supplementary Tables S2 and S3, Supplementary Figure S1).

The guidance for physical activity practice recommends engaging in 150 min of MVPA per week. Videos were available on YouTube, accessible through QR codes included in participants’ educational materials. Exercise videos were shared through WhatsApp^®^ to facilitate home practice.

The three-month intervention followed a structured calendar of topics designed to support lifestyle changes, delivered through group sessions led by trained facilitators. These sessions encouraged goal setting, peer discussion, and strategies to overcome barriers using motivational and problem-solving techniques. Participants received a printed workbook containing educational content, tracking tools for diet and physical activity, and space for notes and reflections (Supplementary Figure S2). Further details on the session plan, group meeting structure, and participant workbook are available in Supplementary Methods S2 (Supplementary Table S4).

Participants in the intervention group attended weekly sessions over a three-month period, alternating between in-person and phone-based meetings. These sessions followed a structured curriculum focused on lifestyle change, including goal setting, problem-solving strategies, and ongoing support. In contrast, the UT group received a single initial session and one final follow-up consultation. The UT group also received guidance on lifestyle modification, with recommendations based on official materials from the Brazilian Ministry of Health. These included the Dietary Guidelines for the Brazilian Population [22] for healthy eating and the Physical Activity Guidelines for the Brazilian Population [21] for increasing weekly MVPA. Both resources are widely promoted for use in the Brazilian primary care setting.

To ensure fidelity and consistency of interventions implementation across all study sites, we developed a standardized operations manual detailing both the protocol procedures and the intervention content. All facilitators and planners participated in a centralized two-day training session prior to study initiation, covering both theoretical foundations and practical aspects of the intervention delivery. Additionally, we held weekly virtual meetings throughout the study period to address questions and provide continuous support regarding the structured lifestyle intervention. These measures were crucial to maintaining the integrity and uniformity of the interventions across the diverse geographic regions involved.

### 2.5. Data Collection

At baseline, all participants completed a standardized questionnaire to collect demographic, clinical, and lifestyle data, covering variables such as age, race, marital status, educational level, income, smoking status, and regular medication use. Socioeconomic and educational data were assessed according to the Brazilian Criteria for Economic Classification [25]. The physical activity was assessed at baseline and at three months by applying the short form International Physical Activity Questionnaire (IPAQ-sf) translated and validated into Portuguese [26].

To evaluate diet quality, we used the mean intake from the two 24 h recalls at each time point. Nutrient values were adjusted for total energy intake using the energy-adjustment method proposed by Willett et al. [27]. Diet quality was assessed using the Diet Quality Index Revised for the Brazilian Population (DQIR) [28,29]. The DQIR comprises twelve components: nine food groups (total fruits; whole fruits; total vegetables; dark green, orange vegetables and legumes; total cereals; whole cereals; milk and dairy products; meats, eggs and legumes; oils), two nutrients (saturated fat and sodium), and one component reflecting energy intake from solid fats, added sugars, and alcohol (SoFAAS). The total DQIR score ranges from 0 to 100, with higher scores indicating better overall dietary quality.

Adherence to the healthy diet proposed in the PROVEN-DIA program was assessed using the BALANCE Index [30]. The index comprises four components corresponding to the BALANCE food groups (green, yellow, blue, and red), each scored from 0 to 10, resulting in a total score ranging from 0 to 40. Higher scores indicate greater adherence to a healthy and cardioprotective dietary pattern, as proposed by the BALANCE guidelines [23]. For the green group, a score of 10 was assigned when all recommended portions were consumed, and 0 when there was no intake. For the yellow group, a score of 10 was given when intake matched the recommendation exactly, and 0 if it deviated by 50% or more. For the blue group, a score of 10 was assigned when intake was equal to or less than the recommendation, and 0 when it exceeded the recommendation by two or more servings. For the red group (ultra-processed foods), a score of 10 indicated no consumption, and 0 was assigned if intake reached or exceeded four servings per day. Intermediate intakes were scored proportionally. The total BALANCE Index score reflects the extent to which individuals adhere to a dietary pattern designed to support cardiovascular health and chronic disease prevention.

Anthropometric (weight, height, waist circumference, and body mass index), blood pressure and biochemical data (fasting blood glucose, serum insulin, and HbA1c) were collected according to standardized procedures at baseline and three months. Insulin resistance was estimated using the Homeostatic Model Assessment for Insulin Resistance (HOMA-IR), calculated as [fasting glucose (mg/dL) × fasting insulin (µU/mL)]/405 [31].

To ensure consistency and accuracy in data management, a specific data monitoring plan was designed. All researchers received training to uniformly collect data using a standardized case report form (CRF), available in both electronic and printed formats. The electronic CRF was registered on REDCap^®^, which facilitated data monitoring and helped prevent missing or erroneous information. Research teams were instructed to monitor adverse events and serious adverse events (SAEs) during a 3-month follow-up. SAEs were reported to the Ethics Committee within 48 h, while other adverse events were also recorded and reported in the final study report to the Ethics Committee.

### 2.6. Outcome Measures

The primary objective of this study was to evaluate the feasibility of implementing a structured lifestyle intervention in individuals at high risk for T2D. Feasibility was assessed through participants’ adherence to behavioral changes, which reflects both their understanding of the intervention and their ability to incorporate the recommended habits into daily life. Specifically, the study aimed to promote improvements in diet quality and increases in weekly physical activity, two modifiable behaviors with well-established roles in reducing T2D risk. Accordingly, the primary outcome was defined as a composite indicator: an improvement of at least 1 point in the BALANCE Index red group score (reflecting healthier eating behavior) and achieving 150 min or more of moderate-to-vigorous physical activity (MVPA) per week at the 3-month follow-up. This threshold was selected based on prior evidence from a randomized controlled trial showing that similar magnitude changes in BALANCE scores were associated with improvements in total diet quality, BMI, and waist circumference among individuals in secondary prevention for cardiovascular disease [32]. Additionally, both components—weekly MVPA and BALANCE Index score—were also assessed individually as continuous variables to capture the magnitude of lifestyle changes. Both outcomes were selected to reflect meaningful lifestyle change and to inform the potential scalability of the intervention in broader public health settings. Other outcomes measured include diet quality measured by DQIR, fasting blood sugar, HbA1c, and body weight mean at 3 months.

### 2.7. Statistical Plan

For the present study, a total sample size of 220 participants (110 per group) was estimated. This sample would provide 90% power to detect a between-group difference of 50 min in mean weekly physical activity time [33] and a 1-point difference in the mean red score of the BALANCE Index [32], assuming a 5% significance level and an anticipated loss to a follow-up of 20%. Categorical patient characteristics were described using absolute and relative frequencies, while continuous variables were summarized as means and standard deviations. For the primary outcome (adherence to the BALANCE Index plus MVPA), we employed a mixed-effects regression model with fixed effects for group, time, and their interaction, and adjusted for sex and research center. No imputation methods were applied. Analyses were conducted based on available case data, with no replacement of missing values. A sensitivity analysis using the imputed data was conducted to assess the robustness of the primary findings. To address missing data during the 3-month follow-up, we performed multiple imputations using the *mice* package in R. This approach identifies variables with missing values and fits appropriate statistical models for each. For binary variables, logistic regression models were applied. Five imputed datasets (m = 5) were generated, and the estimates from each dataset were combined using Rubin’s rules via the *pool* function. The adjustment for sex was necessary due to a significant baseline imbalance between groups. Patients were treated as random effects to account for within-subject correlation over time. For continuous outcomes, the model used an identity link function, while for binary outcomes, a logit link function was applied. Sensitivity analyses were performed among study completers using the same model structure and covariate adjustments. All analyses were conducted using R version 4.2.1, with the “lme4” package, employing the lmer function for continuous outcomes and glmer for binary outcomes. A two-sided significance level of 5% was adopted for all hypothesis tests.

## 3. Results

**Figure 1:**
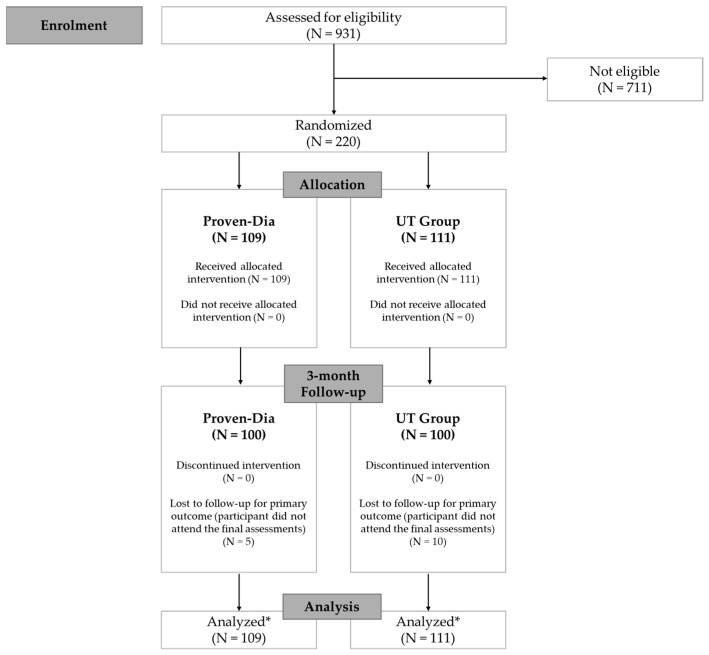
Study flowchart. * Data analysis was based on available case data, but participants lost to follow-up were retained in the statistical analysis according to the intention-to-treat principle.

Eligibility was evaluated in a total of 931 individuals recruited from five centers, each located in one of Brazil’s geographic regions. A total of 220 participants were enrolled and followed until October 2023. Of these, 205 (93.2%) completed the study. The 15 participants who did not complete follow-up were either lost to contact—despite multiple attempts via telephone, email, and family members—or declined to participate in the final assessments (Figure 1).

Baseline characteristics were overall comparable between groups, except for sex, with a predominance of women (71.8%), particularly in the intervention group (78.9% vs. 64.9% in the control group; *p* = 0.03) (Table 1). The study population had a mean age of 48.7 ± 9.6 years. Most participants were married or in a stable union (62.7%), and 40.9% self-identified as mixed race. More than half reported a monthly household income below USD 1012,53. At baseline, the mean BMI was 33.5 ± 6.1 kg/m^2^ and the mean waist circumference was 102.6 ± 13.2 cm, indicating a population with a high prevalence of obesity and central adiposity. Glycemic parameters reflected a high-risk profile for diabetes (mean fasting glucose: 101.1 ± 16.9 mg/dL; HbA1c: 5.7 ± 0.5%). Use of chronic medications was common: 44.5% used antihypertensives, 16.4% antidiabetics, and 15% lipid-lowering agents. Overall, baseline characteristics were similar between groups, except for the higher proportion of women in the intervention group.

There were no significant differences in baseline characteristics between participants who completed the follow-up and those who did not, except for race. A higher proportion of White participants discontinued the study, whereas a greater proportion of mixed-race participants (classified as “pardo” in Brazilian demographic terms) remained in the trial. (Supplementary Table S5). Among those who completed the protocol, the intervention group had a higher baseline BMI compared to the control group (*p* = 0.038) (Supplementary Table S6).

Regarding the primary outcome—defined as a simultaneous improvement in diet quality and an increase in weekly MVPA—25 participants (25%) in the PROVEN-DIA group and 31 participants (31%) in the usual treatment (UT) group met both criteria at the 3-month follow-up (Table 2). No statistically significant difference was observed between groups (*p* = 0.29), suggesting similar levels of behavioral adherence during the intervention period.

**Figure 2:**
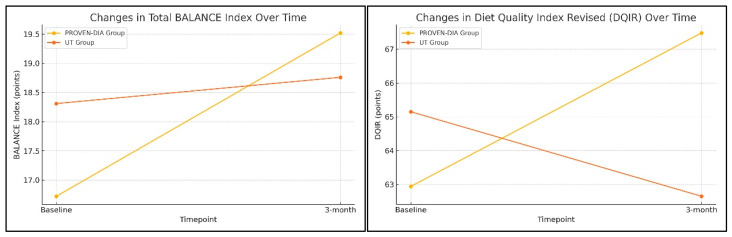
Changes in dietary quality indicators after the intervention: BALANCE Index and DQIR scores at baseline and 3-month follow-up in the Proven-Dia and UT groups.

However, when analyzed separately, improvements in diet quality were significantly more pronounced in the Proven-Dia group. The total BALANCE Index increased by 2.8 points in the Proven-Dia group, compared to only 0.5 points in the control group (*p* = 0.03). This improvement was particularly driven by higher adherence to recommendations related to the blue food group (animal protein), with significantly better scores in the intervention group at three months (*p* = 0.04) (Figure 2). In terms of overall dietary quality, a significant group-by-time interaction was observed for the DQIR (β = 6.81; *p* < 0.001) (Figure 2)., indicating a meaningful improvement in the Proven-Dia group relative to the control. Specifically, the Proven-Dia group improved from 62.94 (±14.39) to 67.48 (±14.39), whereas the control group declined from 65.15 (±14.47) to 62.65 (±14.73). The improvement in IQDR was primarily driven by increased vegetable consumption (*p* = 0.014), along with reductions in total saturated fat (*p* = 0.003) and added sugar intake (*p* = 0.032). Despite these qualitative improvements in dietary patterns, no significant differences were detected in total energy intake or macronutrient composition between groups (Table 3).

To assess the potential impact of missing data on the primary outcome, we conducted a sensitivity analysis. The imputed analysis yielded slightly different rates of adherence, with 19% in the PROVEN-DIA group and 26% in the UT group achieving simultaneous improvement in both diet quality and physical activity at 3 months (*p* = 0.21). These results were consistent with the complete case analysis and support the robustness of our findings. In addition, analyses presented in Supplementary Table S7, show that both groups improved their BALANCE Index scores, with a significantly greater increase in the total score in the intervention group (*p* = 0.04). The PROVEN-DIA group also showed a significantly greater improvement in the blue food group score (*p* = 0.02) and in the Revised Diet Quality Index (DQIR, *p* = 0.01).

In contrast, no significant changes were observed in physical activity levels. The proportion of participants achieving at least 150 min of MVPA per week remained stable and it was similar between groups after follow-up (23.3% in Proven-Dia vs. 27.9% in UT; *p* = 0.40). Likewise, the total MVPA (min/week) did not differ between groups at follow-up (*p* = 0.70).

Regarding clinical parameters, no significant differences were observed between groups in fasting glucose, HbA1c, or body weight over the three-month follow-up period.

A total of 47.7% of participants (*n* = 105/220) reported at least one adverse event during the study period. None of the events was serious or related to the intervention. Adverse events were more frequently reported in the Proven-Dia group (60.9%, *n* = 64) than in the UT group (39.1%, *n* = 41). The most common events in the Proven-Dia group were minor infections (including allergies, flu, and COVID-19; 31.2%, *n* = 20), while the UT group more frequently reported changes in glycemic biomarkers (43.9%, *n* = 18).

## 4. Discussion

This pilot randomized trial aimed to assess the feasibility of implementing a structured lifestyle intervention tailored to the Brazilian public health system for individuals at high risk for T2D. Feasibility was evaluated based on behavioral adherence, measured through changes in diet quality and weekly time spent in moderate-to-vigorous physical activity. After three months, overall adherence—defined as simultaneous improvement in both diet and physical activity behaviors—was similar between the intervention and control groups. However, when analyzed separately, participants in the PROVEN-DIA group showed greater improvement in dietary quality. Importantly, the significant improvement in diet quality observed over just three months is itself a meaningful achievement. Although no significant changes were observed in total energy intake or macronutrient composition, the intervention group demonstrated meaningful improvements in dietary quality. This apparent contradiction highlights that enhancements in food choices—such as increasing vegetable consumption and reducing added sugars—can positively impact diet quality without substantially altering macronutrient distribution. The carbohydrates from added sugars may have been replaced by those from vegetables, resulting in a similar overall proportion but a nutritionally superior profile. This phenomenon has also been observed in other dietary interventions, where improvements in diet quality indices occurred independently of nutrient-level changes, underscoring the importance of evaluating food patterns beyond macronutrient composition alone [34].

A growing body of evidence demonstrates that even short-term improvements in dietary patterns are associated with substantial health benefits, including reductions in cardiovascular risk, better glycemic control, and improvements in overall quality of life [35]. Specifically, diets richer in minimally processed foods, fruits, vegetables, and adequate sources of protein—as promoted in the BALANCE framework—are consistently linked to lower risks of diabetes progression and cardiometabolic diseases [36,37,38]. These improvements in diet quality may have a preventive effect, even before measurable changes in clinical biomarkers such as HbA1c or weight occur, especially in high-risk populations.

We hypothesized that the intervention would lead to superior outcomes in both diet and physical activity, based on the structured and supportive format of the PROVEN-DIA program, which included weekly contact and culturally adapted educational materials. Previous studies, such as the original Diabetes Prevention Program (DPP) and its international adaptations [10,11,12,13,14,15,16] have demonstrated significant improvements in lifestyle behaviors through similar interventions. A Brazilian study by Sartorelli et al. [39] for instance, showed improved dietary habits and physical activity levels in high-risk women following lifestyle counseling within primary care settings. However, consistent with our findings, other studies in low- and middle-income settings have also reported modest or null effects on physical activity, suggesting structural and contextual barriers to behavioral change beyond health education alone. Notably, the dietary improvements observed in this pilot trial are particularly encouraging from a public health perspective, given the critical need for accessible and low-cost interventions to address the escalating burden of T2D. Supporting individuals in improving diet quality—even in the absence of immediate changes in physical activity—can yield significant health benefits. A robust body of evidence demonstrates that enhanced dietary quality is independently associated with lower risks of T2D, cardiovascular disease, and all-cause mortality [35]. Therefore, refining and expanding the nutritional component of the PROVEN-DIA program holds substantial potential to contribute meaningfully to national diabetes prevention efforts and the promotion of overall population health.

Several factors likely contributed to the lack of improvement in physical activity and should be acknowledged as methodological limitations of the current pilot design. First, participants reported relatively high baseline activity levels, often due to physically demanding occupations (e.g., construction, cleaning), which may have reduced the potential for further measurable gains. Second, the intervention was delivered exclusively by nutritionists, without the direct involvement of physical activity professionals, which may have limited the specificity, emphasis, and personalization of this component. Although physiotherapists contributed to the protocol design, they were not involved in its implementation. This represents a structural limitation in the design of the intervention, rather than merely an issue of delivery. Importantly, this interpretation is supported by previous studies indicating that multidisciplinary teams are more effective in promoting physical activity engagement within lifestyle interventions [40]. In addition, well-established barriers—such as low motivation, lack of safe environments for exercise, and socioeconomic constraints—may have further limited physical activity uptake, particularly in socially vulnerable populations. We acknowledge that these barriers were not sufficiently accounted for in the design of this pilot intervention [41,42,43].

The absence of significant improvements in physical activity and the limited combined behavioral adherence highlights important challenges in the design and implementation of lifestyle interventions within real-world public health settings. Rather than interpreting these findings as shortcomings, they should be seen as critical insights into the structural, contextual, and delivery-related factors that shape behavior change. As a feasibility trial, PROVEN-DIA was intended to identify what works, what does not, and why. The dietary improvements observed confirm the potential of the program’s nutritional component. Conversely, the lack of significant change in physical activity points to the need for stronger engagement strategies, multidisciplinary support, and infrastructure tailored to the realities of underserved populations. These findings do not diminish the relevance of the study; on the contrary, they illuminate the necessary adaptations for a future definitive trial and reinforce the value of feasibility studies in refining complex behavioral interventions.

In addition to the important limitation of the physical activity component of the intervention that we identified, this study has several limitations. The short follow-up period may not have been sufficient to capture sustained behavioral changes. The sample size, calculated for the dietary outcome, may have been underpowered to detect differences in physical activity or clinical markers. Additionally, the unequal distribution of women between groups may have influenced results, although this was adjusted for in the statistical models. Another limitation is that the BALANCE dietary guidance was not provided to the control group, potentially limiting comparability for that outcome. Although no upper limit for BMI or age was established in this pilot study, our results suggest that the absence of these criteria did not impair the feasibility or short-term behavioral outcomes observed. The sample included predominantly middle-aged adults (mean age 48.7 ± 9.6 years) with a mean BMI of 33.5 ± 6.1 kg/m^2^. However, for future large-scale trials with clinical outcomes such as diabetes incidence, defining upper thresholds for BMI and age may be necessary to account for heterogeneity in metabolic response and risk profiles. Another limitation is the score used to identify high-risk individuals. Although the CDC/ADA Diabetes Risk Test has not been formally validated in Brazil, it has been applied to national datasets such as the Brazilian National Health Survey [44]. Nevertheless, the lack of formal validation in the Brazilian context is a limitation of this study.

Despite these limitations, the study has important strengths, including high retention rates, the use of validated instruments to assess diet quality and physical activity, and real-world implementation through public health structures, which enhances external validity. Furthermore, understanding the facilitators and barriers to implementation and adherence—both from the perspective of participants and healthcare providers—will be crucial for refining the PROVEN-DIA program. A complementary qualitative study is planned to explore these factors in depth, aiming to uncover the real-world challenges and enablers related to dietary changes and physical activity engagement. These insights will be instrumental in optimizing the intervention, particularly in strengthening the physical activity component and enhancing nutritional counseling. By identifying context-specific barriers, such as socioeconomic constraints, environmental limitations, motivational challenges, and structural issues within the public health system, the program can be adapted to be more effective, acceptable, and scalable. This patient- and provider-centered approach is essential for the development of sustainable diabetes prevention strategies that are responsive to the needs and realities of the target population.

As a pilot randomized trial, this study was not designed or powered to detect statistically significant differences in clinical outcomes such as HbA1c or diabetes incidence. In accordance with CONSORT guidelines for pilot and feasibility trials, the primary objective was to assess feasibility—particularly, whether participants could adhere to the proposed lifestyle intervention. Thus, the findings are exploratory and should be interpreted with caution. Nevertheless, this pilot phase generated valuable insights that inform improvements in both the intervention content and research procedures for a future definitive trial. Although overall adherence was feasible and encouraging, no improvements were observed in physical activity outcomes, likely due to suboptimal design and delivery of this component. Therefore, key areas for enhancement include involving a multidisciplinary team to strengthen the physical activity support [40] and addressing economic and geographic barriers that may hinder physical activity adherence [41,42,43].

## 5. Conclusions

In this pilot randomized trial, the PROVEN-DIA intervention demonstrated high feasibility and acceptability within the Brazilian public health system. Participants in the intervention group demonstrated significant improvements in diet quality over three months, highlighting the potential of structured, culturally adapted dietary interventions to drive meaningful behavioral change in populations at high risk for T2D. However, no significant changes were observed in physical activity levels or clinical parameters during the study period. These findings suggest that while dietary behavior can be effectively modified in the short term through targeted nutritional counseling, additional strategies may be required to influence broader lifestyle changes. A larger, adequately powered trial with longer follow-up is now warranted to determine the effectiveness of the PROVEN-DIA program in reducing diabetes incidence and improving cardiometabolic health outcomes. These results contribute to the development of scalable, evidence-based diabetes prevention strategies within the Brazilian public health system.

## Figures and Tables

**Table 1:** Baseline characteristics of the study participants (*n* = 220).

Variables	Proven-Dia Group (*n* = 109)	UT Group (*n* = 111)	Total (*n* = 220)
Sex (female), *n* (%)	86 (78.9)	72 (64.9)	158 (71.8)
Age (years), mean (SD)	47.8 ± 9.9	49.6 ± 9.2	48.7 ± 9.6
Marriage status, *n* (%)			
Married	72 (66.1)	66 (59.5)	138 (62.7)
Unmarried, divorced, separated, or widowed	37 (33.9)	45 (40.5)	82 (37.3)
Race and ethnicity, *n* (%)			
White	35 (32.1)	29 (26.1)	64 (29.1)
Black	29 (26.6)	34 (30.6)	63 (28.6)
Asian	0 (0.0)	3 (2.7)	3 (1.4)
Mixed race (Parda)	45 (41.3)	45 (40.5)	90 (40.9)
Household income category (USD /month) α, *n* (%)			
≥USD 3840.03	8 (7.3)	14 (12.6)	22 (10)
>USD 3840.03 and ≥USD 1822.92	15 (13.8)	17 (15.3)	32 (14.5)
>USD 1822.92 and ≥USD 1012.53	39 (35.8)	36 (32.4)	75 (34.1)
>USD 1012.53 and ≥USD 576.48	22 (20.2)	18 (16.2)	40 (18.2)
>USD 576.48 and ≥USD 345.86	15 (13.8)	19 (17.1)	34 (15.5)
<USD 345.86	10 (9.2)	7 (6.3)	17 (7.7)
Years of Schooling, *n* (%)			
>14 years	41 (37.6)	49 (44.1)	90 (40.9)
12–14 years	45 (41.3)	39 (35.1)	84 (38.2)
9–11 years	12 (11)	9 (8.1)	21 (9.5)
5–8 years	8 (7.3)	11 (9.9)	19 (8.6)
<5 years	3 (2.8)	3 (2.7)	6 (2.7)
Alcohol consumption (g/d), mean ± SD	12.4 ± 21.6	9.4 ± 13.5	10.7 ± 17.6
Current smoker, *n* (%)	16 (14.7)	19 (17.1)	35 (15.9)
Body weight (kg), mean ± SD	89.3 ± 18	88.1 ± 17.4	88.7 ± 17.7
BMI (kg/m^2^), mean ± SD	34.2 ± 6.4	32.8 ± 5.7	33.5 ± 6.1
Waist circumference (cm), mean ± SD	104 ± 13.8	101.2 ± 12.5	102.6 ± 13.2
SBP (mmHg), mean ± SD	124.8 ± 17.9	125.1 ± 17.6	125 ± 17.7
DBP (mmHg), mean ± SD	82.5 ± 13.5	79.9 ± 10.0	81.2 ± 11.9
Fasting blood glucose (mg/dL), mean ± SD	101.4 ± 19.1	100.9 ± 14.6	101.1 ± 16.9
HbA1c (%), mean ± SD	5.6 ± 0.5	5.7 ± 0.5	5.7 ± 0.5
Insulin (µUI/mL), mean ± SD	12.5 ± 7.7	12 ± 6.7	12.2 ± 7.2
Use of chronic medications, *n* (%)			
Antidiabetics	17 (15.6)	19 (17.1)	36 (16.4)
Lipid-lowering agents	12 (11.0)	21 (18.9)	33 (15.0)
Antihypertensives	44 (40.4)	54 (48.6)	98 (44.5)
Antithrombotics	2 (1.8)	9 (8.1)	11 (5.0)

BMI: Body Mass Index; HbA1c: Glycated hemoglobin; SBP: Systolic Blood Pressure; DBP: Diastolic Blood Pressure; UT: usual treatment. α Values based on dollar exchange rate from 30 April 2024 (USD 1.00 = BRL 5.6840).

**Table 2:** Primary and secondary outcomes according to Proven-Dia and UT groups.

Outcomes	Proven-Dia Group		UT Group		*p*-Value *
	Baseline (*n* = 109)	3-Month Follow-Up (*n* = 100)	Baseline (*n* =111)	3-Month Follow-Up (*n* = 100)	
BALANCE Indexes					
Green group (points), mean ± SD	4.86 ± 2.55	5.65 ± 2.54	4.91 ± 2.22	5.08 ± 2.72	0.11
Yellow group (points), mean ± SD	3.16 ± 2.65	3.24 ± 2.59	3.74 ± 2.6	3.23 ± 2.52	0.17
Blue group (points), mean ± SD	4.59 ± 3.73	5.37 ± 3.90	5.33 ± 3.53	4.89 ± 3.51	0.04
Red group (points), mean ± SD	4.12 ± 3.23	5.26 ± 3.15	4.34 ± 3.32	5.56 ± 3.38	0.87
Total score (points), mean ± SD	16.72 ± 5.97	19.52 ± 6.12	18.31 ± 5.95	18.76 ± 6.68	0.03
DQIR, mean ± SD	62.94 ± 14.39	67.48 ± 14.39	65.15 ± 14.47	62.65 ± 14.73	<0.001
Physical activity ^α^, *n* (%)	26 (23.8)	24 (23.3)	25 (22.5)	26 (27.9)	0.40
Total MVPA, mean ± SD	102.69 ± 199.92	103.27 ± 192.01	101.76 ± 224.08	117.69 ± 240.82	0.70
Body weight (kg), mean ± SD	89.29 ± 18.04	87.12 ± 15.68	88.06 ± 17.45	85.58 ± 15.49	0.22
Fasting blood glucose (mg/dL), mean ± SD	101.38 ± 19.13	102.57 ± 15.32	100.87 ± 14.56	101.68 ± 14.64	0.89
HbA1c (%), mean ± SD	5.23 ± 1.56	5.53 ± 0.49	5.24 ± 1.65	5.54 ± 0.48	0.91

DQIR: Diet Quality Index Revised for the Brazilian Population; MVPA: Moderate to vigorous physical activity; UT: usual treatment. * *p*-values correspond to the group-by-time interaction, adjusted for sex and research center. ^α^ Achieving, at least, 150 min/week of moderate to vigorous physical activity.

**Table 3:** Nutrient intake before and after the lifestyle intervention in the PROVEN-DIA and control groups.

Outcomes	Proven-Dia Group		UT Group		*p*-Value *
	Baseline (*n* = 109)	3-Month Follow-Up (*n* = 100)	Baseline (*n* =111)	3-Month Follow-Up (*n* = 100)	
Energy (kcal)	1605.48 ± 99.33	1484.84 ± 83.33	1613.41 ± 45.47	1490.28 ± 57.87	0.840
Total carbohydrate (g)	187.86 ± 35.05	185.26 ± 40.67	170.67 ± 33.49	185.26 ± 40.67	0.610
Carbohydrate (%)	46.05 ± 9.3	45.94 ± 8.41	46.52 ± 8.34	45.53 ± 10.07	0.600
Total fiber (g)	20.13 ± 9.32	20.97 ± 8.91	20.54 ± 9.13	20.05 ± 9.44	0.420
Total protein (g)	77.97 ± 19.95	75.21 ± 15.92	80.62 ± 20.59	78.75 ± 22.11	0.860
Protein (%)	19.48 ± 5.00	20.35 ± 4.57	20.03 ± 5.26	21.20 ± 6.26	0.800
Total fat (g)	61.40 ± 14.02	55.70 ± 12.89	59.94 ± 11.69	55.14 ± 12.86	0.700
Fat (%)	34.46 ± 7.70	33.71 ±7.29	33.44 ± 6.49	33.28 ± 7.52	0.700
Saturated (g)	20.84 ± 5.32	18.15 ± 5.06	19.67 ± 5.44	18.45 ± 5.48	0.150
Monounsatured (g)	19.48 ± 6.38	17.51 ± 5.50	18.48 ± 5.69	17.70 ± 6.08	0.290
Polyunsaturated (g)	12.71 ± 4.97	12.73 ± 3.86	13.75 ± 4.18	12.75 ± 3.80	0.170

UT: usual treatment. * *p*-values correspond to the group-by-time interaction, adjusted for sex and research center. Data adjusted for total energy intake using the energy-adjustment method [27].

## Data Availability

The data are not publicly available due to privacy. Data and materials will be made available upon reasonable request to the corresponding author, following the completion of a specific form provided by Beneficência Portuguesa de São Paulo and in accordance with the institution’s data sharing policies.

## Supplementary Materials

The following supporting information can be downloaded at: https://www.mdpi.com/article/10.3390/nu17152518/s1, Supplementary Table S1. CONSORT 2010 checklist of information to include when reporting a pilot or feasibility trial; Supplementary Methods S1. Dietary Intervention Based on the Brazilian Cardioprotective Nutritional Program (BALANCE); Supplementary Figure S1. Brazilian Cardioprotective Diet visual approach; Supplementary Table S2. The number of portions per day according to BALANCE food groups; Supplementary Table S3. Examples of region-specific foods according to BALANCE food groups; Supplementary Methods S2. Intervention Structure and Educational Materials (PROVEN-DIA Program); Supplementary Table S4. Intervention description per session of the PROVEN-DIA Program; Supplementary Figure S2. Participant workbook; Supplementary Table S5. General characteristics of the study participants according to follow-up; Supplementary Table S6. Baseline characteristics between participants who complete the study according to UT and Proven-Dia groups. Supplementary Table S7. Sensitivity analysis of primary and secondary outcomes in the PROVEN-DIA and usual treatment (UT) groups after multiple imputations.
