# Supplementary material for: A Multicenter Pilot Randomized Trial of a Lifestyle Intervention to Prevent Type 2 Diabetes in High-Risk Individuals
Source: Nutrients. 2025 Jul 31;17(15):2518. doi: 10.3390/nu17152518 (PMC12348132; doi:10.3390/nu17152518)
Supplement: Supplementary file 1 [file nutrients-17-02518-s001.zip › nutrients-3755113-supplementary.pdf]

A Multicenter Pilot Randomized Trial of a Lifestyle Intervention to Prevent Type 2 Diabetes in High-Risk Individuals

Supplementary Materials

Item	Title / Description	Page(s)
Table S1	CONSORT 2010 Checklist for Pilot or Feasibility Trials	2
Supplementary Methods S1	Dietary Intervention Based on the Brazilian Cardioprotective Nutritional Program (BALANCE)	5
Figure S1	Brazilian Cardioprotective Diet visual approach	5
Table S2	Number of portions per day according to BALANCE food groups	6
Table S3	Examples of region-specific foods according to BALANCE food groups	7
Supplementary Methods S2	Intervention Structure and Educational Materials (PROVEN-DIA Program)	9
Table S4	Description of PROVEN-DIA Intervention Sessions	9
Figure S2	Participant Workbook	12
Table S5	General characteristics of the study participants according to follow-up	13
Table S6	Baseline characteristics between participants who complete the study according to UT and Proven-Dia groups	15
Table S7	Sensitivity analysis of primary and secondary outcomes in the PROVEN-DIA and usual treatment (UT) groups after multiple imputations.	17

Supplementary Tables

Table S1. CONSORT 2010 checklist for Pilot or Feasibility Trials

Section/Topic	Item No	Checklist item	Reported on page No
**Title and abstract**			
	1a	Identification as a pilot or feasibility randomised trial in the title	1
	1b	Structured summary of pilot trial design, methods, results, and conclusions (for specific guidance see CONSORT abstract extension for pilot trials)	1,2
**Introduction**			
Background and objectives	2a	Scientific background and explanation of rationale for future definitive trial, and reasons for randomised pilot trial	2,3
	2b	Specific objectives or research questions for pilot trial	3
**Methods**			
Trial design	3a	Description of pilot trial design (such as parallel, factorial) including allocation ratio	3,4
	3b	Important changes to methods after pilot trial commencement (such as eligibility criteria), with reasons	Not applicable
Participants	4a	Eligibility criteria for participants	3,4
	4b	Settings and locations where the data were collected	3
	4c	How participants were identified and consented	3
Interventions	5	The interventions for each group with sufficient details to allow replication, including how and when they were administered	4-6
Outcomes	6a	Completely defined prespecified assessments or measurements to address each pilot trial objective specified in 2b, including how and when they were assessed	6,7
	6b	Any changes to pilot trial assessments or measurements after the pilot trial commenced, with reasons	Not applicable
	6c	If applicable, prespecified criteria used to judge whether, or how, to proceed with future definitive trial	7,8
Sample size	7a	Rationale for numbers in the pilot trial	7

	7b	When applicable, explanation of any interim analyses and stopping guidelines	Not applicable
Randomisation:			
Sequence generation	8a	Method used to generate the random allocation sequence	4
	8b	Type of randomisation(s); details of any restriction (such as blocking and block size)	4
Allocation concealment mechanism	9	Mechanism used to implement the random allocation sequence (such as sequentially numbered containers), describing any steps taken to conceal the sequence until interventions were assigned	4
Implementation	10	Who generated the random allocation sequence, who enrolled participants, and who assigned participants to interventions	4
Blinding	11a	If done, who was blinded after assignment to interventions (for example, participants, care providers, those assessing outcomes) and how	4
	11b	If relevant, description of the similarity of interventions	4
Statistical methods	12	Methods used to address each pilot trial objective whether qualitative or quantitative	7,8
**Results**			
Participant flow (a diagram is strongly recommended)	13a	For each group, the numbers of participants who were approached and/or assessed for eligibility, randomly assigned, received intended treatment, and were assessed for each objective	8
	13b	For each group, losses and exclusions after randomisation, together with reasons	8
Recruitment	14a	Dates defining the periods of recruitment and follow-up	8
	14b	Why the pilot trial ended or was stopped	Not applicable
Baseline data	15	A table showing baseline demographic and clinical characteristics for each group	9
Numbers analysed	16	For each objective, number of participants (denominator) included in each analysis. If relevant, these numbers should be by randomised group	9
Outcomes and estimation	17	For each objective, results including expressions of uncertainty (such as 95% confidence interval) for any estimates. If	9-11

		relevant, these results should be by randomised group	
Ancillary analyses	18	Results of any other analyses performed that could be used to inform the future definitive trial	11
Harms	19	All-important harms or unintended effects in each group (for specific guidance see CONSORT for harms)	11
	19a	If relevant, other important unintended consequences	Not applicable
**Discussion**			
Limitations	20	Pilot trial limitations, addressing sources of potential bias and remaining uncertainty about feasibility	13
Generalisability	21	Generalisability (applicability) of pilot trial methods and findings to future definitive trial and other studies	14
Interpretation	22	Interpretation consistent with pilot trial objectives and findings, balancing potential benefits and harms, and considering other relevant evidence	12-14
	22a	Implications for progression from pilot to future definitive trial, including any proposed amendments	14
**Other information**			
Registration	23	Registration number for pilot trial and name of trial registry	3
Protocol	24	Where the pilot trial protocol can be accessed, if available	Not applicable
Funding	25	Sources of funding and other support (such as supply of drugs), role of funders	15
	26	Ethical approval or approval by research review committee, confirmed with reference number	15

Reference:

Eldridge SM, Chan CL, Campbell MJ, Bond CM, Hopewell S, Thabane L, Lancaster GA; PAFS consensus group. CONSORT 2010 statement: extension to randomised pilot and feasibility trials. Pilot Feasibility Study. 2016 Oct 21;2:64. doi: 10.1186/s40814-016-0105-8.

Supplementary Methods S1 – Dietary Intervention Based on the Brazilian Cardioprotective Nutritional Program (BALANCE)

Classification of Food Groups

All foods and preparations included in the Brazilian Cardioprotective Diet are categorized into four color-coded groups, inspired by the colors of the Brazilian flag. This visual approach was designed to help guide healthier food choices based on both nutritional quality and recommended frequency of consumption [1,2]:

- ***Green group:*** Foods encouraged for frequent consumption, such as fruits, vegetables, legumes, and skim milk. Green represents abundance and health — and, as in the flag, is the most prominent color in the diet.
- ***Yellow group:*** Foods to be eaten in moderation, including whole grains, nuts, and vegetable oils.
- ***Blue group:*** Foods recommended in limited portions, such as lean meats, eggs, and low-fat cheeses.
- ***Red group:*** Ultra-processed foods, which are discouraged in the program. Red is not present in the national flag, symbolizing that these foods should not be part of a cardioprotective diet.

References:

1. Brasil. Ministério da Saúde. Alimentação Cardioprotetora: manual de orientações para os profissionais de saúde da Atenção Básica / Ministério da Saúde, Hospital do Coração. – Brasília: Ministério da Saúde, 2018. 138 p. : il.
2. Weber B et al. Implementation of a Brazilian Cardioprotective Nutritional (BALANCE) Program for improvement on quality of diet and secondary prevention of cardiovascular events: A randomized, multicenter trial. Am Heart J. 2019 Sep;215:187-197. doi: 10.1016/j.ahj.2019.06.010.

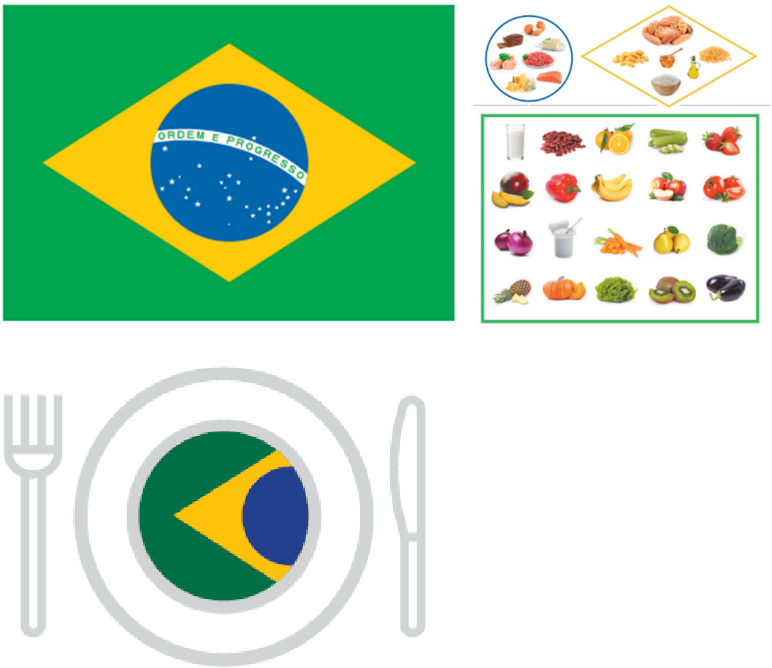

Figure S1. Brazilian Cardioprotective Diet visual approach.

Nutritional Criteria for Food Classification

The classification of foods into the green, yellow, blue, or red groups in the Brazilian Cardioprotective Diet is based on their nutrient density, using specific cut-off values per gram. Foods were assessed according to their energy density (≤ 1.11 kcal/g), saturated fat (≤ 0.01 g/g), cholesterol (≤ 0.04 mg/g), and sodium content (≤ 2.01 mg/g). Items that met all or nearly all of these criteria were placed in the green group, indicating high nutritional quality and suitability for frequent consumption. Foods that exceeded one or two of the thresholds were classified as yellow, while those surpassing three or four limits were placed in the blue group, reflecting the need for more restricted intake. Ultra-processed foods, regardless of their nutrient profile, were assigned to the red group and are discouraged altogether.

Daily Energy Prescription

The total daily caloric intake in the Brazilian Cardioprotective Diet is defined based on the individual's nutritional goal. For weight maintenance, the recommended intake is 25 kcal per kilogram of body weight per day. For individuals aiming for weight loss, the target is reduced to 20 kcal/kg/day, while for those requiring weight gain, the recommendation is set at 30 kcal/kg/day or more. Once the total caloric requirement is established, it guides the calculation of the number of daily servings from each food group, ensuring that nutrient density and dietary balance are maintained according to the diet's structure.

Menu Planning and Portion Guidelines

Participants received individualized meal plans tailored to their energy requirements, which ranged from 1,400 to 2,400 kcal/day in 200-kcal increments. These caloric needs were determined based on each participant's body weight and nutritional goal: 20 kcal/kg/day for weight loss, 25 kcal/kg/day for weight maintenance, and ≥30 kcal/kg/day for weight gain. Once the total daily energy requirement was established, the number of recommended servings from each BALANCE food group was calculated accordingly. Each caloric level was aligned with the original nutritional goals of the BALANCE program, particularly with respect to saturated fat and sodium limits, while also adapting macronutrient distribution to restrict total carbohydrate intake to 50–60% of daily energy. The table below summarizes the number of daily servings per food group according to caloric level:

Table S2. Number of portions per day according to BALANCE food groups.

Calorie Plan (kcal/day)	Green portions/day	Yellow portions/day	Blue portions/day	Red
1400	8	4	2	Avoid
1600	9	5	2	Avoid
1800	10	5	3	Avoid
2000	11	6	3	Avoid
2200	12	6	4	Avoid
2400	15	13	4	Avoid

Adaptation for Diabetes Prevention

To adapt the BALANCE Program for diabetes prevention, all original menus—originally developed for cardiovascular risk reduction—were revised to ensure appropriate carbohydrate control in line with dietary recommendations for normoglycemic individuals. The objective was to limit total daily carbohydrate intake to 45–48% of total calories and fat intake to 20–35%, while preserving the core principles of the BALANCE Program: the proportional distribution among food groups (green, yellow, and blue) and the emphasis on simplicity and cultural relevance for practical implementation.

The menu adaptations were guided by several strategies:

- Reducing carbohydrate-dense foods from the yellow group—especially refined grains—as originally proposed in the BALANCE Program, replacing them with

protein-rich foods from the green and blue groups, such as beans, skimmed dairy, lean meats, and low-fat fish.

- Prioritizing low glycemic index carbohydrates, including whole fruits, legumes, and high-fiber foods.
- Rebalancing portion sizes to maintain nutritional adequacy while reducing glycemic impact.
- Using a standard caloric prescription of 20 kcal/kg/day, reflecting the goal of weight loss in individuals at high risk for diabetes.
- Ensuring an even distribution of meals and snacks throughout the day, promoting better glycemic control and satiety.

A total of 87 menus were reformulated—each representing a typical day's meals—covering all caloric ranges from 1,400 to 2,400 kcal/day, in 200-kcal increments. For each caloric level, at least 3 to 7 menus were developed per region, reflecting regional dietary habits and food preferences across Brazil's five geographic regions. These revised menus maintained the cultural and practical strengths of the original BALANCE program while aligning with current recommendations for diabetes prevention.

Table S3. Examples of region-specific foods according to BALANCE food groups.

**Brazilian region**	**Green group**	**Yellow group**	**Blue group**
Midwest	Black beans, green *papaya*, pumpkin *curau*, okra salad, *jabuticaba*	*Farofa de banana*	Baked fish fillet, bread pudding, cornmeal cake, chicken with *pequi*, *chipa*
Northeast	Coconut water, cashew, *caja* mango	Couscous with milk, *cassava* flour, coconut candy and taro	Green corn cake, fish stew with shrimp, *cassava* flour, *bolo de rolo* and *acarajé*
North	Cashew, red guava, soursop	Couscous with milk, *tapioca*, plantain, *cassava* flour, *jambu* rice	*Cupuaçu* cream, *tucunaré* stew, *maniçoba*, *tacacá* and chicken with *tucupi*
Southeast	Carioca beans, corn with okra	French bread	Carrot cake, Paulista-type couscous, cheese bread, *tropeiro*

Brazilian region	Green group	Yellow group	Blue group
			beans, *bolinho de chuva,* Minas-type cheese
South	fig	Sagu grape, capeletti soup, caramelized pumpkin	Chicken stew with corn, *pirão*, bread pudding, *cuca*

Supplementary Methods S2 – Intervention Structure and Educational Materials (PROVEN-DIA Program)

The PROVEN-DIA intervention lasted 3 months and was composed of structured group sessions designed to promote sustainable lifestyle changes. Each session followed a predefined content agenda, focusing nutritional and physical activity components related to diabetes prevention.

Group Dynamics

Group sessions were led by trained professionals who encouraged participants to set realistic goals and discuss barriers and facilitators to behavior change. The facilitator applied motivational interviewing principles and problem-solving strategies to support individual and group progress.

Educational Calendar and Session Topics

The program followed a session-by-session educational agenda. Examples of covered topics included:

- Understanding diabetes risk and prevention
- Building healthier meals using BALANCE principles
- Increasing physical activity in daily life
- Coping with stress and emotional eating
- Setting goals and maintaining motivation

Table S4. Description of PROVEN-DIA Intervention Sessions.

Session	Interval in days after randomization	Brief intervention description
1st individual session	Day of randomization	Additional data collection followed by goal setting, educational materials delivery, presentation of holistic gymnastics exercises and detailed explanations about the program
1st phone call by the facilitator (up to 15 minutes)	5-9 days	Tracking the achievement of goals and facilitating adherence to the program by strengthening the connection between participant and professional
1st group session	10-20 days	Promotion of critical thinking and encouragement to share experiences, knowledge and perceptions about

Session	Interval in days after randomization	Brief intervention description
(1-hour session with 3-15 participants)		healthy eating while introducing the BALANCE principles based on Brazilian flag strategy
2nd phone call by the facilitator (up to 15 minutes)	20-24 days	Tracking the achievement of goals and facilitating adherence to the program alongside with motivational strategies
2nd individual session	25-35 days	Explanation of the BALANCE portion sizes and goals adaptation, when needed
3rd phone call by the facilitator (up to 15 minutes)	35-39 days	Tracking and celebrating the achievement of goals alongside coping with barriers
2nd group session (1-hour session with 3-15 participants)	40-50 days	Sharing challenges and barriers faced throughout lifestyle change in order to seek solutions by formulating 5 step action plans
4th phone call by the facilitator (up to 15 minutes)	58-63 days	Tracking the achievement of goals and facilitating adherence to the program by strengthening the connection between participant and professional
3rd group session (1-hour session with 3-15 participants)	70-80 days	Educational strategies for healthy foods recognition based on healthy and cardioprotective plate strategy
5th phone call by the facilitator (up to 15 minutes)	80-84 days	Checking the daily application of the healthy and cardioprotective plate through photo sharing alongside with food consumption collection and celebration of 3-month program engagement
3rd individual session	85-95 days	Data collection and encouragement for keeping a more active and healthier lifestyle followed by the celebration of not only the news habits achieved, the overcoming of barriers and difficulties, but also the improvement in quality of life and well-being

Participant Workbook

All participants in the intervention group received a printed workbook containing:

- Educational content aligned with each session's topic
- Sections for goal-setting and self-monitoring
- Tables to record daily food intake and physical activity
- Reflection spaces for questions and personal notes

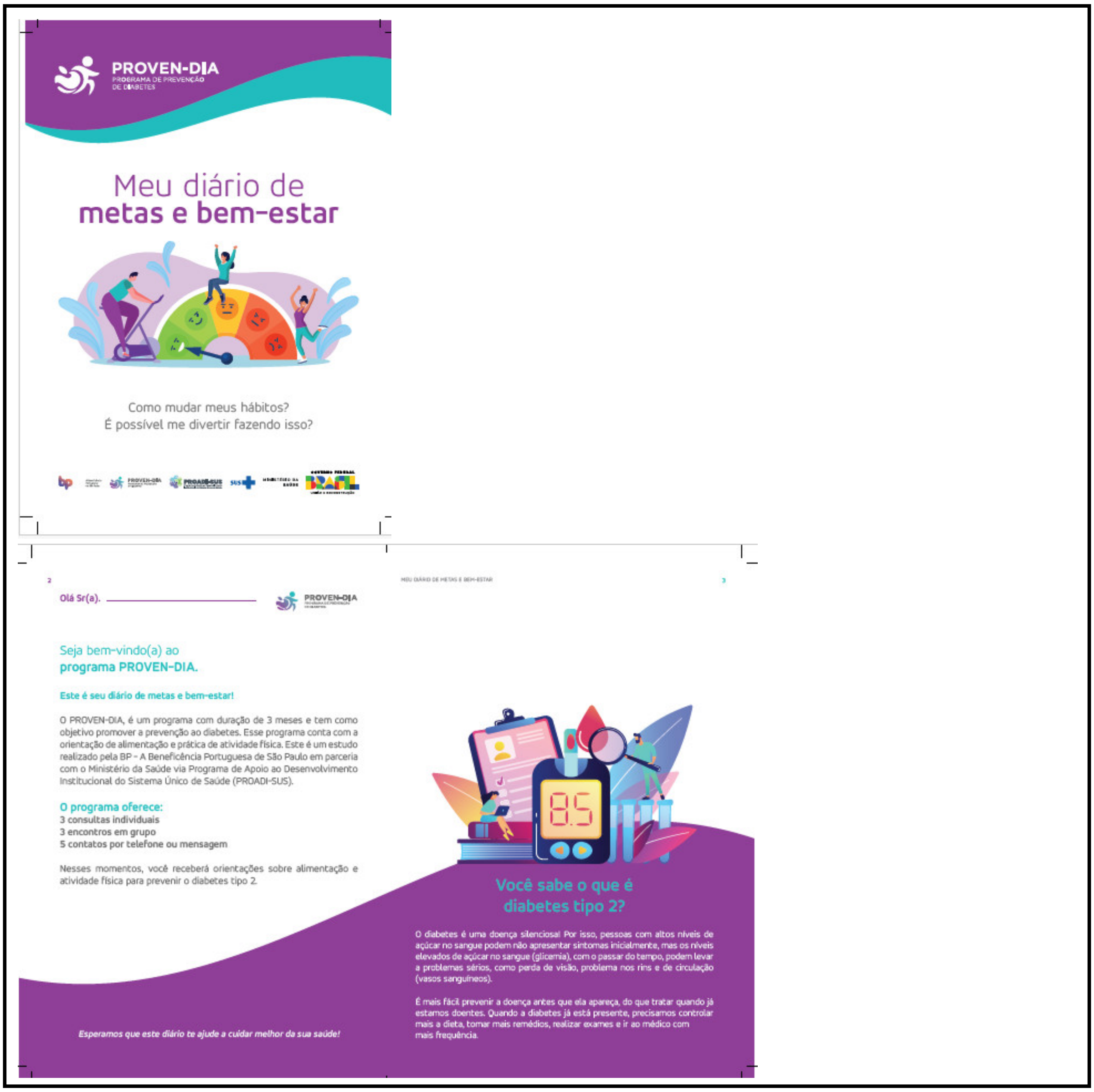
PROVEN-DIA

Meu diário de
metas e bem-estar

Como mudar meus hábitos?
É possível me divertir fazendo isso?

2

Olá Sr(a). ______________

Seja bem-vindo(a) ao
programa PROVEN-DIA.

Este é seu diário de metas e bem-estar!

O PROVEN-DIA, é um programa com duração de 3 meses e tem como objetivo promover a prevenção ao diabetes. Esse programa conta com a orientação de alimentação e prática de atividade física. Este é um estudo realizado pela BP - A Beneficência Portuguesa de São Paulo em parceria com o Ministério da Saúde via Programa de Apoio ao Desenvolvimento Institucional do Sistema Único de Saúde (PROADI-SUS).

O programa oferece:
3 consultas individuais
3 encontros em grupo
5 contatos por telefone ou mensagem

Nesses momentos, você receberá orientações sobre alimentação e atividade física para prevenir o diabetes tipo 2.

Esperamos que este diário te ajude a cuidar melhor da sua saúde!

3

Você sabe o que é
diabetes tipo 2?

O diabetes é uma doença silenciosa! Por isso, pessoas com altos níveis de açúcar no sangue podem não apresentar sintomas inicialmente, mas os níveis elevados de açúcar no sangue (glicemia), com o passar do tempo, podem levar a problemas sérios, como perda de visão, problema nos rins e de circulação (vasos sanguíneos).

É mais fácil prevenir a doença antes que ela apareça, do que tratar quando já estamos doentes. Quando a diabetes já está presente, precisamos controlar mais a dieta, tomar mais remédios, realizar exames e ir ao médico com mais frequência.

Figure S2. Participant workbook.

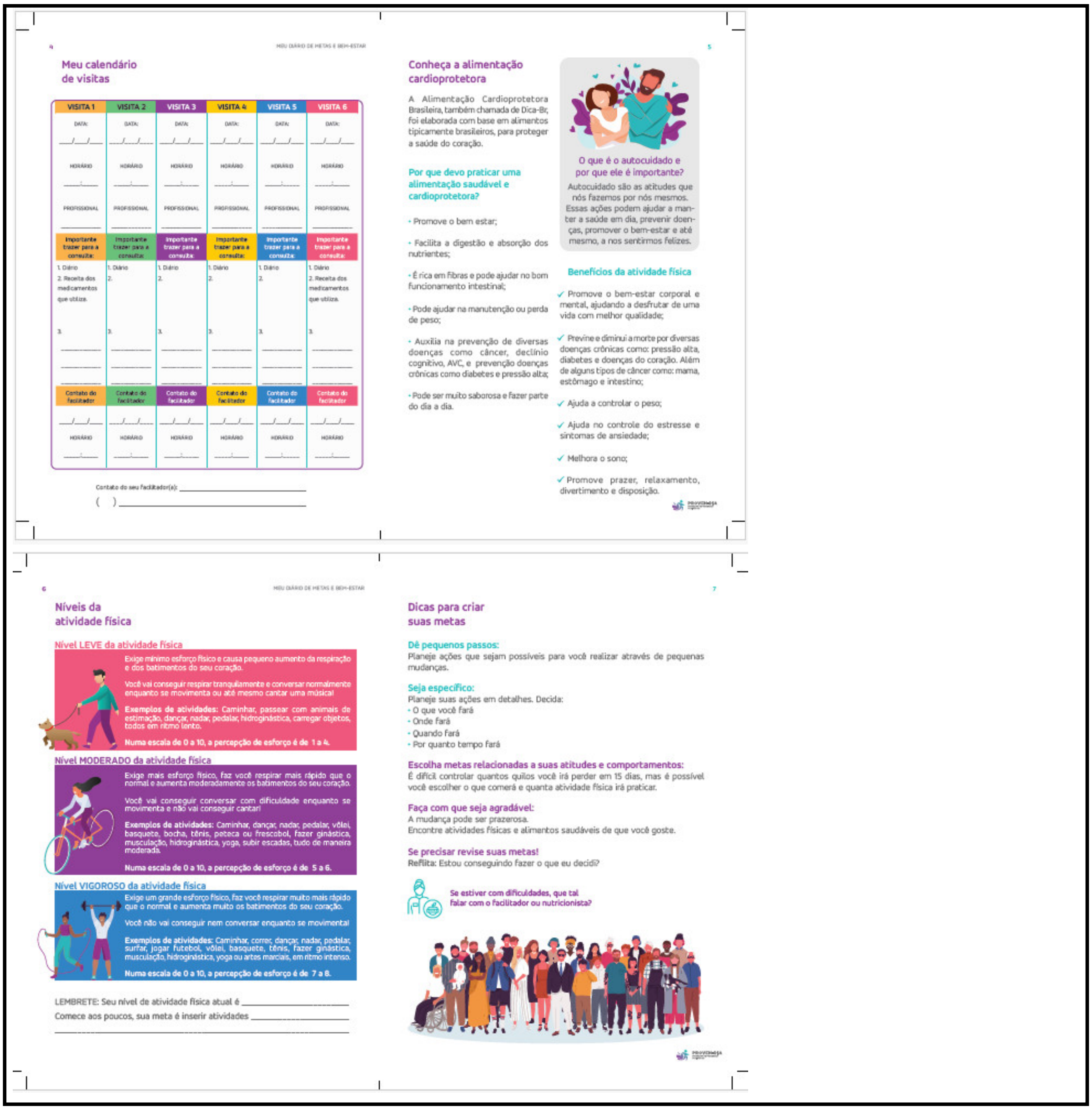

4 MEU DIÁRIO DE METAS E BEM-ESTAR

Meu calendário de visitas

VISITA 1	VISITA 2	VISITA 3	VISITA 4	VISITA 5	VISITA 6
DATA: ___/___/___	DATA: ___/___/___	DATA: ___/___/___	DATA: ___/___/___	DATA: ___/___/___	DATA: ___/___/___
HORÁRIO ___:___	HORÁRIO ___:___	HORÁRIO ___:___	HORÁRIO ___:___	HORÁRIO ___:___	HORÁRIO ___:___
PROFISSIONAL ________	PROFISSIONAL ________	PROFISSIONAL ________	PROFISSIONAL ________	PROFISSIONAL ________	PROFISSIONAL ________
Importante trazer para a consulta:	Importante trazer para a consulta:	Importante trazer para a consulta:	Importante trazer para a consulta:	Importante trazer para a consulta:	Importante trazer para a consulta:
1. Diário 2. Receita dos medicamentos que utiliza. 3. ________	1. Diário 2. 3. ________	1. Diário 2. 3. ________	1. Diário 2. 3. ________	1. Diário 2. 3. ________	1. Diário 2. Receita dos medicamentos que utiliza. 3. ________
Contato do facilitador	Contato do facilitador	Contato do facilitador	Contato do facilitador	Contato do facilitador	Contato do facilitador
___/___/___ HORÁRIO ___:___	___/___/___ HORÁRIO ___:___	___/___/___ HORÁRIO ___:___	___/___/___ HORÁRIO ___:___	___/___/___ HORÁRIO ___:___	___/___/___ HORÁRIO ___:___

Contato do seu facilitador(a): ______________________

(   ) ______________________

5

Conheça a alimentação cardioprotetora

A Alimentação Cardioprotetora Brasileira, também chamada de Dica-Br, foi elaborada com base em alimentos tipicamente brasileiros, para proteger a saúde do coração.

Por que devo praticar uma alimentação saudável e cardioprotetora?

- Promove o bem estar;
- Facilita a digestão e absorção dos nutrientes;
- É rica em fibras e pode ajudar no bom funcionamento intestinal;
- Pode ajudar na manutenção ou perda de peso;
- Auxilia na prevenção de diversas doenças como câncer, declínio cognitivo, AVC, e prevenção doenças crônicas como diabetes e pressão alta;
- Pode ser muito saborosa e fazer parte do dia a dia.

O que é o autocuidado e por que ele é importante?

Autocuidado são as atitudes que nós fazemos por nós mesmos. Essas ações podem ajudar a manter a saúde em dia, prevenir doenças, promover o bem-estar e até mesmo, a nos sentirmos felizes.

Benefícios da atividade física

- ✓ Promove o bem-estar corporal e mental, ajudando a desfrutar de uma vida com melhor qualidade;
- ✓ Previne e diminui a morte por diversas doenças crônicas como: pressão alta, diabetes e doenças do coração. Além de alguns tipos de câncer como: mama, estômago e intestino;
- ✓ Ajuda a controlar o peso;
- ✓ Ajuda no controle do estresse e sintomas de ansiedade;
- ✓ Melhora o sono;
- ✓ Promove prazer, relaxamento, divertimento e disposição.

6 MEU DIÁRIO DE METAS E BEM-ESTAR

Níveis da atividade física

Nível LEVE da atividade física

Exige mínimo esforço físico e causa pequeno aumento da respiração e dos batimentos do seu coração.

Você vai conseguir respirar tranquilamente e conversar normalmente enquanto se movimenta ou até mesmo cantar uma música!

Exemplos de atividades: Caminhar, passear com animais de estimação, dançar, nadar, pedalar, hidroginástica, carregar objetos, todos em ritmo lento.

Numa escala de 0 a 10, a percepção de esforço é de 1 a 4.

Nível MODERADO da atividade física

Exige mais esforço físico, faz você respirar mais rápido que o normal e aumenta moderadamente os batimentos do seu coração.

Você vai conseguir conversar com dificuldade enquanto se movimenta e não vai conseguir cantar!

Exemplos de atividades: Caminhar, dançar, nadar, pedalar, vôlei, basquete, bocha, tênis, peteca ou frescobol, fazer ginástica, musculação, hidroginástica, yoga, subir escadas, tudo de maneira moderada.

Numa escala de 0 a 10, a percepção de esforço é de 5 a 6.

Nível VIGOROSO da atividade física

Exige um grande esforço físico, faz você respirar muito mais rápido que o normal e aumenta muito os batimentos do seu coração.

Você não vai conseguir nem conversar enquanto se movimenta!

Exemplos de atividades: Caminhar, correr, dançar, nadar, pedalar, surfar, jogar futebol, vôlei, basquete, tênis, fazer ginástica, musculação, hidroginástica, yoga ou artes marciais, em ritmo intenso.

Numa escala de 0 a 10, a percepção de esforço é de 7 a 8.

LEMBRETE: Seu nível de atividade física atual é ______________________

Comece aos poucos, sua meta é inserir atividades ______________________

____

7

Dicas para criar suas metas

Dê pequenos passos:
Planeje ações que sejam possíveis para você realizar através de pequenas mudanças.

Seja específico:
Planeje suas ações em detalhes. Decida:
- O que você fará
- Onde fará
- Quando fará
- Por quanto tempo fará

Escolha metas relacionadas a suas atitudes e comportamentos:
É difícil controlar quantos quilos você irá perder em 15 dias, mas é possível você escolher o que comerá e quanta atividade física irá praticar.

Faça com que seja agradável:
A mudança pode ser prazerosa.
Encontre atividades físicas e alimentos saudáveis de que você goste.

Se precisar revise suas metas!
Reflita: Estou conseguindo fazer o que eu decidi?

Se estiver com dificuldades, que tal falar com o facilitador ou nutricionista?

Figure S2. Participant workbook.

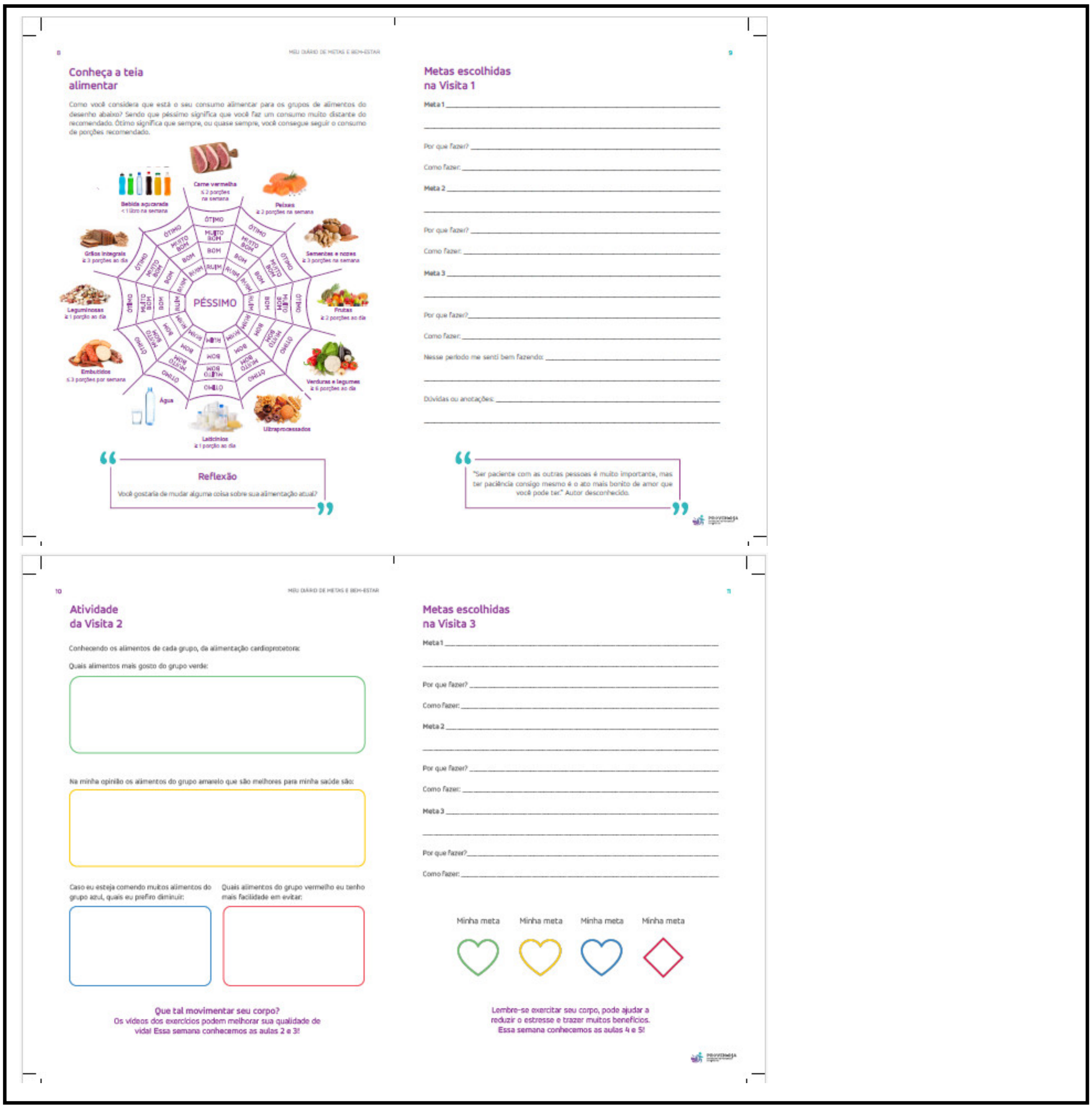

Figure S2 (continued). Participant workbook.

12 MEU DIÁRIO DE METAS E BEM-ESTAR

Exemplos de alimentos da dieta cardioprotetora

1 porção de alimento do grupo verde equivale a, por exemplo:

Hortaliças cruas ou cozidas -> 2 pegadores ou 4 colheres de sopa
Legumes crus, cozidos ou refogados -> 4 colheres de sopa
Feijão -> 4 colheres de sopa ou 1 concha
Leite desnatado ou iogurte desnatado -> 1 copo de requeijão
Leite semidesnatado -> 1/2 copo de requeijão
Iogurte desnatado ou light -> 1 pote individual
Frutas:
Banana ou laranja ou Mexerica -> 1 unidade média
Mamão papaia -> 1/2 unidade
Abacaxi ou Melancia ou Melão -> 1 fatia média

1 porção de alimento do grupo amarelo equivale a, por exemplo:

Arroz branco ou integral -> 1 colher de arroz ou 2 colheres de sopa
Macarrão cozido -> 4 colheres de sopa
Pão francês -> 1/2 unidade ou 1 unidade sem miolo
Pão caseiro branco ou integral -> 1 fatia
Farinha de mandioca -> 1 colher de sopa
Aveia -> 2 colheres de sopa
Leite integral -> 1/2 copo de requeijão
Geleia de frutas com açúcar -> 2 colheres de chá
Castanhas (castanha de caju, amendoim, etc): 2 colheres de sopa

1 porção de alimento do grupo amarelo equivale a, por exemplo:

Carne de boi ou carne de porco >1 bife pequeno (100g)
Frango e Peixe -> 1 bife médio (120g)
Ovo -> 1 unidade
Queijo (branco ou ricota) -> 1 fatia média (30g)
Muçarela -> 1 fatia
Manteiga -> 1 colher de chá
Bolo simples ou recheado -> 1 fatia fina

13

Atividade da Visita 4

Bem-vindo aos 5 passos para superar desafios!

Aqui é um espaço para você identificar os desafios envolvidos na adesão das suas metas para melhorar o seu estilo de vida.

Vamos juntos trabalhar passo a passo para você alcançar suas metas!

Passo 1: Identificar o desafio

Descreva em detalhes um desafio que te impede de aderir às suas metas. O que me impede de aderir à(s) minha(s) meta(s), de mudar meu estilo de vida? Qual o meu principal desafio para aderir à(s) meta(s)? Quais as ações, comportamentos, pensamentos e sentimentos que levam para que eu tenha esse desafio?

____
____

Pense sobre quais opções você tem para superar esse desafio que elencou. Como faço para superar todas as ações, sentimentos e pensamentos envolvidos nesse desafio?

____
____
____

Passo 2: Listar todas as opções para enfrentar o desafio

Liste as ações, comportamentos, pensamentos e sentimentos que levam ao desafio:	Quais opções tenho para solucionar cada ação, comportamento, sentimento e pensamento envolvido no desafio?

14 MEU DIÁRIO DE METAS E BEM-ESTAR

Passo 3: Escolher a melhor opção

Escolha uma das opções que te faça sentido e pareça fácil e tranquila de adotar. Sinto que vou conseguir executar essa opção? Quais são os prós e os contras dessa opção? Por que essa opção é a mais fácil para eu aderir no momento?

Escreva aqui o que você vai fazer:

Fale um pouco sobre porque escolheu essa opção:______________________________

Passo 4: Organizar um plano para executar a opção escolhida

Faça um plano de ação positivo!

Eu vou: ______________________________

Quando vou fazer? ______________________________

O que precisa ser feito antes? ______________________________

O que me ajuda a fazer?

- ______________________________
- ______________________________
- ______________________________

O que pode dar errado?	O que vou fazer se der errado?

Passo 5: Tente! Veja pontos que podem te ajudar a monitorar o plano que você estabeleceu.

Meu plano de ação funcionou?______________________________
Se não, o que não funcionou?______________________________
O que eu posso fazer diferente na próxima vez?______________________________

Lembre-se, é importante nos próximos 15 dias fazer os exercícios da aula 6. Após esses 15 dias, iniciar os exercícios da aula 7 e fazer até o próximo encontro.

15

Atividade da Visita 5

Meu prato saudável

O que eu considero um prato saudável?

____
____
____

Monte seu prato saudável e cardioprotetor referente à sua refeição principal do dia:

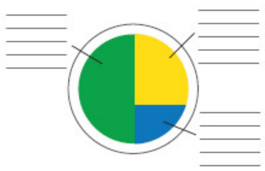

Vamos traduzir suas metas atuais de alimentação para a alimentação cardioprotetora:

Minha meta	Minha meta de alimentação cardioprotetora
Exemplo: Comer mais frutas	Exemplo: Comer mais porções do grupo verde

O que está me mantendo motivado até aqui:______________________________

Lembre-se exercitar seu corpo, traz muitos benefícios.
Até a próxima consulta foque na aula 8.

Figure S2 **(continued).** Participant workbook.

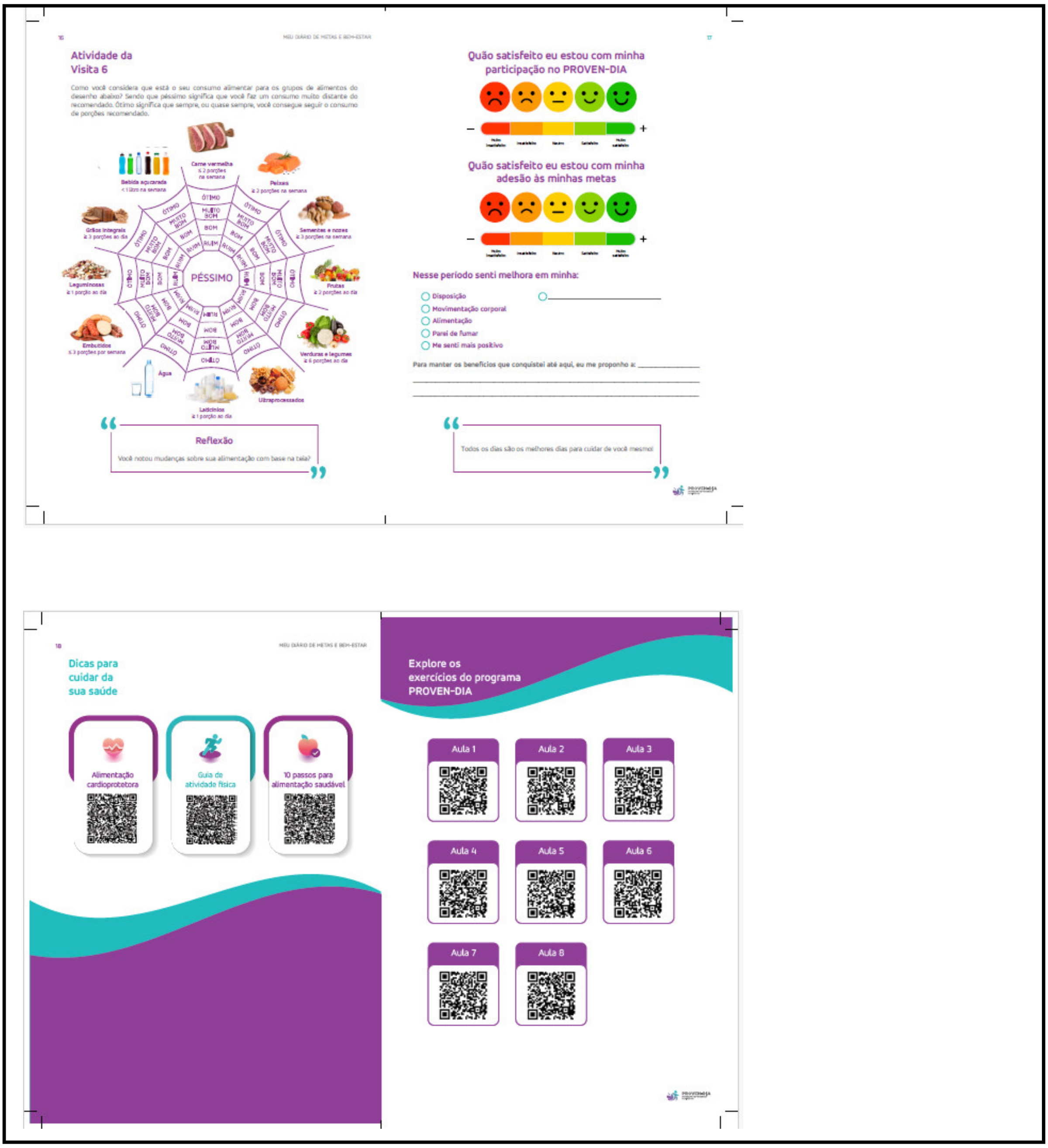

Figure S2 (continued). Participant workbook.

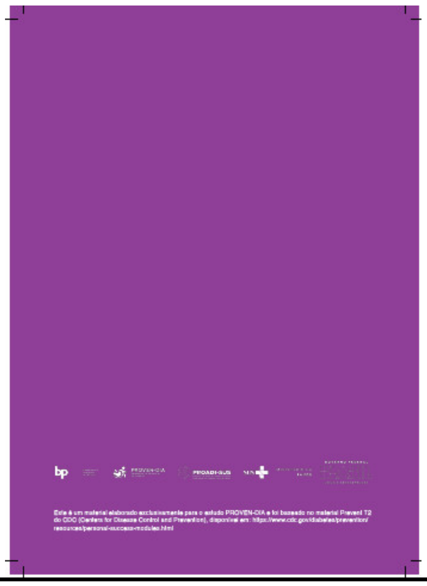

Figure S2 (continued). Participant workbook.

Implementation Materials

To promote fidelity to the intervention model, all researchers followed a standardized guide for planning and conducting each session. A Standardized Operating Procedure (SOP) was also available to ensure a set of step-by-step instruction regarding recruitment, data collection and intervention, among other study aspects. In addition, research teams were trained both prior and alongside the study, including REDCap® and Vivanda systems training, biorepository best practices for research and clinical investigations, revision of Good Clinical Practice (GCP), ethical procedures training emphasizing informed consent and adverse events. Weekly meetings were also conducted to support recruitment, participant inclusion, time-effective problem-solving, and data quality. When applicable, other educational materials were offered in order to overcome challenges and difficulties found throughout the study.

Supplementary Table S5. General characteristics of the study participants according to follow-up.

Variables	Participants who completed follow-up (n= 205)	Participants who lost follow-up (n = 15)	p-value
Sex (female), no./total no. (%)	148/205 (72.2%)	10/15 (66.7%)	0.8712
Age (years), mean ± SD	49.2 ± 9.4	41.3 ± 9.1	0.0024
Marriage status, no./total no. (%)			
Married	131/205 (63.9%)	7/15 (46.7%)	0.3547
Unmarried, divorced, separated, or widowed	74/205 (31.1%)	8/15 (53.3%)	
Race and ethnicity, n (%)			0.001
White	27,8% (57/205)	46,7% (7/15)	
Black	28,8% (59/205)	26,7% (4/15)	
Asian	0,5% (1/205)	13,3% (2/15)	
Mixed race (Parda)	42,9% (88/205)	13,3% (2/15)	
Household income category (USD/month)α, no./total no. (%)			
≥ USD 3840.03	21/205 (10.2%)	1/15 (6.7%)	0.7208
> USD 3840.03 and ≥ U$1822.92	29/205 (14.1%)	3/15 (20.0%)	
> USD1822.92 and ≥ U$ 1012.53	71/205 (34.6%)	4/15 (26.7%)	
> USD 101253 and ≥ U$ 576.48	36/205 (17.6%)	4/15 (26.7%)	
> USD 576.48 and ≥ U$ 345.86	31/205 (15.1%)	3/15 (20.0%)	
≤ USD 345.86	17/205 (8.3%)	0/15 (0.0%)	
Years of Schooling, no./no. total (%)			
> 14 years	86/205 (42%)	4/15 (26.7%)	0.1452
12-14 years	79/205 (38.5%)	5/15 (33.3%)	
9-11 years	17/205 (8.3%)	4/15 (26.7%)	
5-8 years	18/205 (8.8%)	1/15 (6.7%)	
< 5 years	5/205 (2.4%)	1/15 (6.7%)	
Alcohol consumption (g/d), no./total no.(mean ± SD)	136/205 (10.9 ± 18.1)	11/15 (9.2 ± 10.5)	0.9881
Current smoker, no./total no.(%)	33/205 (16.1%)	2/15 (13.3%)	1.0000
Body weight (kg), mean ± SD	88.1 ± 17.4	96.3 ± 20.2	0.0639
BMI (kg/m²), mean ± SD	33.4 ± 6.2	35.2 ± 5.4	0.0834
Waist circumference (cm), mean ± SD	102.3 ± 12.7	107.2 ± 19	0.3082
SBP (mmHg), mean ± SD	124.8 ± 17.9	127.8 ± 15.2	0.4680
DBP (mmHg), mean ± SD	81.1 ± 12.1	81.8 ± 9.0	0.6572

Fasting blood glucose (mg/dL), no./total no. (mean ± SD)	192/205 (101.1 ± 16.9)	13/15 (101.3 ± 17.8)	0.6653
HbA1c (%),no./total no. (mean ± SD)	191/205 (5.7 ± 0.5)	12/15 (5.7 ± 0.4)	0.9555
Insulin (μUI/mL), no./total no. (mean ± SD)	190/205 (12.1 ± 7.2)	11/15 (13.9 ± 7.6)	0.3761
Use of chronic medications, no./total no. (%)			
Antidiabetics	35/205 (17.1%)	1/15 (6.7%)	0.5503
Lipid-lowering agents	31/205 (15.1%)	2/15 (13.3%)	1.0000
Antihypertensives	91/205 (44.4%)	7/15 (46.7%)	0.8961
Antithrombotics	10/205 (4.9%)	1/15 (6.7%)	1.0000

BMI: Body Mass Index; HbA1c: Glycated hemoglobin; SBP: Systolic Blood Pressure; DBP: Diastolic Blood Pressure; UT: usual treatment. α Values based on dollar exchange rate from April 30, 2024 (U$1,00 = R$5,6840).

Supplementary Table S6. Baseline characteristics between participants who complete the study according to UT and Proven-Dia groups.

Variables	UT Group (*n* = 101)	Proven-Dia Group (*n* = 104)	p-value
Sex (female), n (%)	65 (64.4%)	83 (79.8%)	0.0207
Age (years), mean (SD)	50.3 ± 9.0	48.2 ± 9.6	0.1101
Marriage status, n (%)			
Married	60 (59.4%)	71 (68.3%)	0.6014
Unmarried, divorced, separated, or widowed	26 (40.6%)	33 (31.7%)	
Race and ethnicity, n (%)			
White	26 (25.7%)	31 (29.8%)	0.7027
Black	30 (29.7%)	29 (27.9%)	
Asian	1 (1.0%)	0 (0.0%)	
Mixed race (Parda)	44 (43.6%)	44 (42.3%)	
Household income category (USD/month)α, n (%)			
≥ USD 3840.03	14 (13.9%)	7 (6.7%)	0.3271
> USD 3840.03 and ≥ USD1822.92	15 (14.9%)	14 (13.5%)	
> USD1822.92 and ≥ USD 1012.53	33 (32.7%)	38 (36.5%)	
> USD 1012.53 and ≥ USD 576.48	14 (13.9%)	22 (21.2%)	
> USD 576.48 and ≥ USD 345.86	18 (17.8%)	13 (12.5%)	
≤ USD 345.86	7 (6.9%)	10 (9.6%)	
Years of Schooling, n (%)			
> 14 years	47 (46.5%)	39 (37.5%)	0.5227
12-14 years	36 (35.6%)	43 (41.3%)	
9-11 years	6 (5.9%)	11 (10.6%)	
5-8 years	10 (9.9%)	8 (7.7%)	
< 5 years	2 (2.0%)	3 (2.9%)	
Alcohol consumption (g/d), mean ± SD	75 (9.4 ± 13.9)	61 (12.7 ± 22.1)	0.3086
Current smoker, n (%)	18 (17.8%)	15 (14.4%)	0.6370
Body weight (kg), mean ± SD	87.3 ± 17.4	88.9 ± 17.5	0.5848
BMI (kg/m^2), mean ± SD	32.5 ± 5.8	34.2 ± 6.4	0.0381
Waist circumference (cm), mean ± SD	101.0 ± 12.83	104.0± 14.0	0.117
SBP (mmHg), no./total no. (mean ± SD)	100 (125.3 ± 17.9)	103 (124.3 ± 18.0)	0.6976
DBP (mmHg), no./total no. (mean ± SD)	100 (79.8 ± 10.1)	103 (82.4 ± 13.8)	0.2411
Fasting blood glucose (mg/dL), no./total no. (mean ± SD)	95 (100.9 ± 14.3)	97 (101.3 ± 19.3)	0.9110
HbA1c (%),no./total no. (mean ± SD)	95 (5.7 ± 0.5)	96 (5.6 ± 0.5)	0.2211
Insulin (μUI/mL), no./total no. (mean ± SD)	93 (11.8 ± 6.6)	97 (12.4 ± 7.7)	0.5597

Use of chronic medications, no./total no. (%)			
Antidiabetics	18 (17.8%)	17 (16.3%)	0.9242
Lipid-lowering agents	19 (18.8%)	12 (11.5%)	0.2083
Antihypertensives	49 (48.5%)	42 (40.4%)	0.3027
Antithrombotics	8 (7.9%)	2 (1.9%)	0.0952

BMI: Body Mass Index; HbA1c: Glycated hemoglobin; SBP: Systolic Blood Pressure; DBP: Diastolic Blood Pressure; UT: usual treatment. α Values based on dollar exchange rate from April 30, 2024 (U$1,00 = R$5,6840).

Supplementary Table S7. Sensitivity analysis of primary and secondary outcomes in the PROVEN-DIA and usual treatment (UT) groups after multiple imputations.

Outcomes	Proven-Dia Group		UT Group		p-value*
	Baseline (*n* = 109)	3-month follow-up (*n* = 109)	Baseline (*n* = 111)	3-month follow-up (*n* = 111)	
BALANCE Indexes					
Green group (points), mean ± SD	4.86 ± 2.55	5.47 ± 2.66	4.91 ± 2.22	5.19 ± 2.79	0.38
Yellow group (points), mean ± SD	3.16 ± 2.65	3.26 ± 2.59	3.74 ± 2.6	3.34 ± 2.55	0.25
Blue group (points), mean ± SD	4.59 ± 3.73	5.46 ± 3.84	5.33 ± 3.53	4.80 ± 3.50	0.02
Red group (points), mean ± SD	4.12 ± 3.23	5.25 ± 3.14	4.34 ± 3.32	5.52 ± 3.36	0.93
Total score (points), mean ± SD	16.72 ± 5.97	19.44 ± 6.09	18.31 ± 5.95	18.85 ± 6.65	0.04
DQIR, mean ± SD	62.94 ± 14.39	67.60 ± 14.30	65.15 ± 14.47	63.65 ± 14.91	0.01
Physical activity$^{\alpha}$, n (%)	167.51 ± 223.72	196.71 ± 264.14	170.86 ± 260.97	200.95 ± 242.97	0.85
Total MVPA, mean ± SD	102.69 ± 199.92	101.72 ± 187.86	101.76 ± 224.08	110.59 ± 224.71	0.80
Body weight (kg), mean ± SD	89.29 ± 18.04	88.19 ± 16.06	88.06 ± 17.45	86.51 ± 15.63	0.77
Fasting blood glucose (mg/dL), mean ± SD	101.38 ± 19.13	102.87 ± 14.95	100.87 ± 14.56	101.29 ± 14.38	0.63
HbA1c (%), mean ± SD	5.23 ± 1.56	5.57 ± 0.48	5.24 ± 1.65	5.54 ± 0.47	0.83

DQIR: Diet Quality Index Revised for the Brazilian Population; MVPA: Moderate to vigorous physical activity; UT: usual treatment. * P-values correspond to the group-by-time interaction, adjusted for sex and research center. α Achieving, at least, 150 min/week of moderate-to-vigorous physical activity.
